# How do teeth erupt?

**DOI:** 10.1038/s41415-024-7609-z

**Published:** 2024-08-09

**Authors:** Daniel Stonehouse-Smith, Laura Ota, Jadbinder Seehra, Jerry Kwok, Catherine Liu, Maisa Seppala, Martyn T. Cobourne

**Affiliations:** 41415128130001 Centre for Craniofacial & Regenerative Biology, Department of Orthodontics, Faculty of Dental, Oral & Craniofacial Sciences, King´s College London, London, UK; 41415128130002 Dental Core Trainee, Guy´s and St Thomas´ NHS Foundation Trust, UK; 41415128130003 Department of Oral Surgery, Guy´s and St Thomas´ NHS Foundation Trust, UK

## Abstract

The development of normal occlusion requires eruptive migration of teeth from their developmental position in the jaw into a functional position within the oral cavity. This process involves significant and coordinated movement in an axial direction and appropriate eruption through the gingival tissues. The mechanisms regulating these developmental events are poorly understood, and teeth retain eruptive potential throughout their lifespan. In recent years, the use of mouse models has helped to elucidate some of the underlying molecular and biological mechanisms of mammalian tooth eruption. Here, we outline our current understanding of tooth eruption mechanisms and discuss their relevance in terms of known human disorders of tooth eruption.

## Key points

- Teeth in the human dentition retain the ability to erupt throughout life but the molecular mechanisms are poorly understood.
- Recent advances in our understanding of how teeth erupt are summarised.
- Genetic disorders associated with failed eruption of the permanent dentition are discussed with clinical examples and supporting evidence for the biological basis of these conditions.

## Introduction

The establishment of normal occlusion requires eruption of the teeth from their developmental position in the jaw bone into a functional position within the oral cavity. This process involves significant axial movement of the tooth, initiated at the right time and requiring both intra-osseous and intra-oral movement. We know that an intact dental follicle^1^^,^^2^ and normal osteoclast function^3^ are absolute requirements for tooth eruption to proceed normally, while, somewhat surprisingly, the periodontal ligament and root seem to be expendable.^4^ The gubernacular cord, which consists of vascular connective tissue and remnants of the dental lamina, has also been postulated to play an important role during eruption of the teeth, although the exact mechanism is not understood.^5^

Current thinking would suggest that the primary eruption mechanism is dependent upon asymmetric bone remodelling around the developing tooth germ; specifically, resorption in the coronal regions and deposition apically.^6^^,^^7^ However, the precise cellular mechanisms regulating this process in both health and disease are also only poorly understood. In recent years, our understanding of the tooth eruption process has improved, particularly the influence of signalling from the enamel organ and dental follicle of developing teeth, and the role of normal osteoclast function.^1^^,^^2^^,^^3^ Teeth in the permanent dentition most commonly fail to erupt due to idiopathic or pathological mechanical obstruction; however, failure in the eruptive mechanism itself can also occur^8^ and may affect one or a number of teeth in either dentition and can be partial or complete.

Several terms have been ascribed to different types of eruption failure affecting the human dentition. Primary retention of permanent teeth is an isolated condition associated with a localised failure of eruption and no other identifiable local or systemic involvement.^8^^,^^9^ Secondary retention involves the unexplained cessation of further eruption after a tooth has penetrated the oral mucosa.^8^^,^^10^Ankylosis can affect any tooth but would normally be diagnosed alongside a history of trauma or developmental pathology. The known association between eruption defects, and a small number of defined syndromic conditions and findings that non-syndromic eruption defects are often seen in families, suggests an important genetic component underlying failure of normal tooth eruption. Here, we discuss current knowledge of the molecular basis of tooth eruption and focus on two disorders of known genetic aetiology associated with disrupted human tooth eruption. We also discuss these conditions in the context of supportive biological evidence from mouse models.

## The molecular biology of tooth eruption

**Fig. 1:**
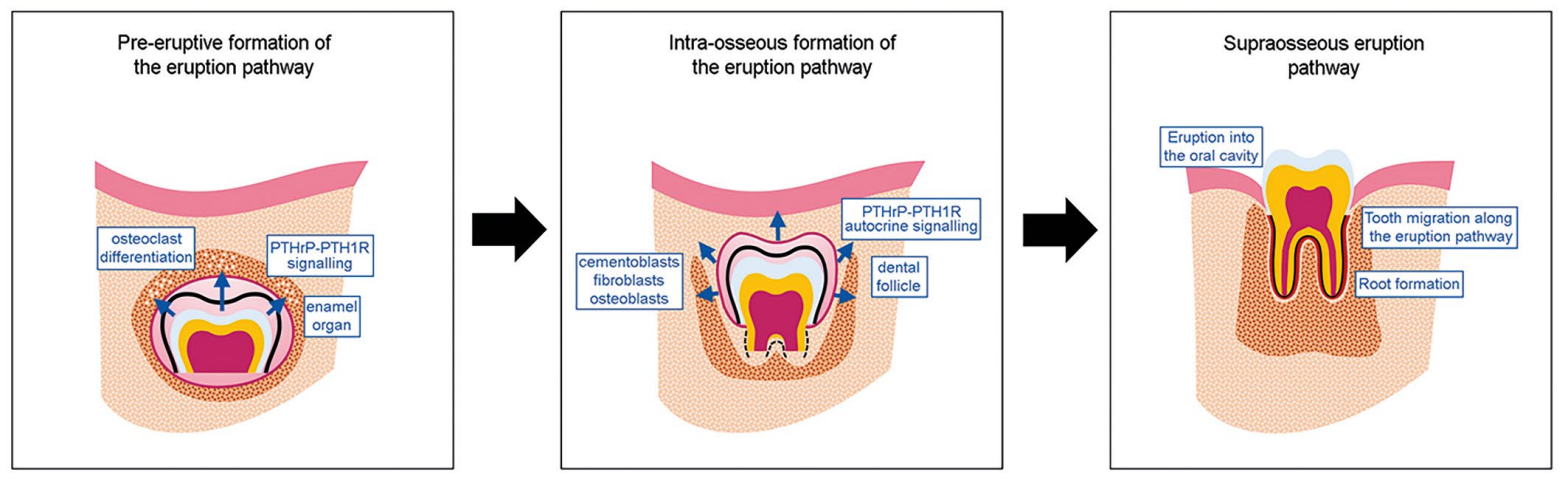
Tooth eruption progresses through three distinct phases: pre-eruptive; intraosseous; and supraosseous

It is now firmly established from experiments in mice that parathyroid hormone-related protein (PTHrP) is not only a key regulator of bone metabolism^11^ but also an essential molecular component of the tooth eruption pathway.^12^ PTHrP is a locally acting peptide released from the enamel organ and dental follicle, which acts through its G-protein-coupled parathyroid hormone 1 receptor (PTH1R) to coordinate the complex process of root formation and eruption.^13^ PTHrP activity in the enamel organ epithelium is initially required for local osteoclast differentiation and alveolar bone resorption around the coronal region of the developing tooth to create an early pre-eruption pathway within the crypt of alveolar bone (Fig. 1). In mice lacking PTHrP function, the teeth remain covered in alveolar bone and fail to erupt; however, genetic restoration of PTHrP signalling in the enamel organ of these mice can rescue the eruption defect through re-establishment of this early eruption pathway.^14^

Normal tooth eruption requires more than just the formation of a pre-eruptive bony channel above the developing tooth and there is evidence that PTHrP signalling within the dental follicle is also essential for establishment of an intraosseous eruption pathway. PTHrP is strongly expressed in the mesenchymal-derived dental follicle and developing root surface of the tooth germ. Sophisticated cell lineage-tracing and loss-of-function studies in PTHrP+ mesenchymal progenitor cells has demonstrated that PTHrP-PTH1R within cell (autocrine) regulation is essential for maintaining appropriate cell fate during the process of tooth eruption.^15^ Specifically, PTHrP+ cells initially localise to the dental follicle but become progressively restricted to cementoblasts, periodontal ligament fibroblasts and osteoblasts within the bony crypt of the developing tooth. Local ablation of PTH1R function in PTHrP+ dental follicle cells before eruption produces a shift in cell fate within this cell population, away from cementoblasts (on the acellular cementum), periodontal ligament cells and alveolar osteoblasts, to a homogenous population of non-physiological cementoblast-like cells, which precociously form cellular cementum on the tooth root surface. This ectopic cementum seems to inhibit eruption, with two-thirds of first molar teeth failing to erupt in mice lacking PTH1R function in dental follicle cells, despite normal PTHrP signalling in the enamel organ.^15^

Osteoclasts themselves are ultimately derived from erythro-myeloid progenitor cells^3^ with their differentiation induced by colony stimulating factor-1 (CSF1) and coordinated through signalling between these osteoclast progenitors and local osteoblasts - specifically, induction by TNF-related receptor activator of nuclear factor-κB ligand (RANKL) signalling and inhibition by osteoprotegerin (OPG). PTHrP promotes the expression of RANKL and downregulates OPG, activating osteoclasts and inducing bone resorption.^3^ In the developing tooth, the dental follicle also plays a key role in this process, releasing both CSF1 and PTHrP.^9^^,^^16^ A clear relationship therefore seems to exist between appropriate osteoclastogenesis, normal bone development and tooth eruption, with impaired development of functional osteoclasts leading to excessive levels of bone and cartilage mineralisation and osteopetrosis, a group of disorders associated with increased bone density and long associated with disrupted tooth eruption. Indeed, mice lacking function of key mediators of osteoclastogenesis have osteopetrosis and failure of tooth eruption.^17^^,^^18^^,^^19^

The molecular analysis of mesenchymal progenitor cell regulation during mouse molar tooth eruption has identified a putative list of potential genes involved in PTHrP-PTH1R signalling during tooth eruption. Gene ontology has revealed potential roles for gene pathways involved with biomineral tissue development, protein secretion, chemotaxis, inflammation and neuron projection development, emphasising the highly coordinated and complex mechanisms associated with tooth eruption.^15^ Collectively, it seems a wide range of processes are required to regulate tooth eruption, which highlights the need for comprehensive assays of gene variation in human subjects with tooth eruption anomalies.

## Primary failure of eruption

**Fig. 2:**
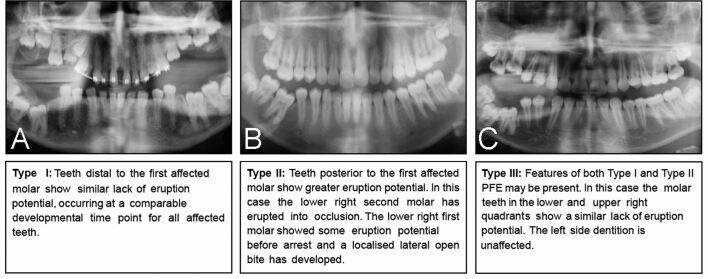
Classification and radiographic clinical features of PFE. a) Type I. b) Type II. c) Type III

The term primary failure of eruption (PFE) has been used to describe a relatively rare and isolated form of tooth eruption failure that predominantly affects the posterior teeth, which often partially erupt and then stop before they can achieve a functional occlusion.^20^ Comprehensive phenotypic analyses of PFE have characterised a variety of features associated with this condition (Table 1).^20^ Indeed, PFE has been further classified into three types based upon the essential clinical features^21^ (Fig. 2).

**Table 1:** Clinical features of human tooth eruption defects with a known genetic basis

Condition (gene)	Clinical features	
Primary failure of eruption (*PTH1R*)	Rare Familial, linked to mutations in *PTH1R* gene Non-ankylosed tooth or teeth; however, attempts at extrusion of the affected teeth results in ankylosis. Teeth do not respond to orthodontic forces; treatment with a continuous wire can worsen the malocclusion by intruding adjacent teeth^43^ Affected tooth or teeth can erupt partially, then cease to erupt further, resulting in infraocclusion Posterior teeth affected Never affects the anterior teeth^44^ Can affect both primary and secondary dentitions^23^ Typically, all teeth distal to the most mesial tooth affected will also be affected^23^ May be unilateral or bilateral	
	**Type I** Progressive open bite from the anterior to posterior of the dental arches Eruption failure occurs at or near the same time for the teeth in the same quadrant	**Type II** Progressive open bite from the anterior to posterior of the dental arches but the teeth distal to the most mesial affected tooth show greater but still inadequate eruption potential A gradient of the time of failure is observed, allowing further eruption of the posterior dentition^21^
	**Type III** Combination of features from both Type I and Type II	
Cleidocranial dysplasia (*RUNX2*)	General skeletal features include short stature, long neck, narrow shoulders, excessive mobility of the shoulder girdle, hypoplastic and/or aplastic clavicles, delayed ossification and small pelvic bones Craniofacial features include prominent forehead, frontal and parietal bossing, wide-set eyes, wide nasal bridge, multiple wormian bones, open fontanelles, delayed ossification of the skull bones, hypoplastic or absent nasal bones. Of dental relevance there is often: Mandibular prognathism Multiple (and late forming) supernumerary teeth Prolonged retention and delayed exfoliation of the primary dentition Multiple unerupted permanent and supernumerary teeth (which may develop dentigerous cysts)^45^

**Fig. 3:**
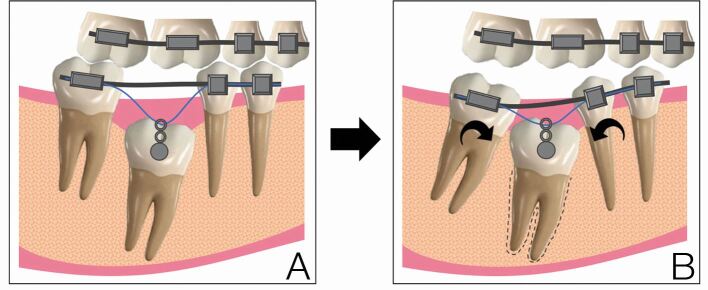
Primary failure of eruption is difficult to manage clinically. a) Orthodontic traction to an affected tooth generally results in ankylosis. b) Prolonged orthodontic traction results in disruption to the occlusion, often causing intrusion of adjacent teeth towards the affected tooth and a worsening lateral open bite

It has been known for some time that mutations in the *PTH1R* gene are a significant cause of autosomal dominant PFE in humans.^22^ Since the original discovery of a link between PFE and *PTH1R* function, more than 50 variants in this gene have been identified in association with PFE.^23^ However, PTH-related signalling currently remains the only known candidate pathway identified in primary eruption failure. The *PTH1R* ablation and cell fate alteration experiments in the mouse are interesting because they provide insight that is consistent with some of the key features associated with PFE; in particular, the ability of the tooth to establish an initial intra-osseous eruption pathway but then stop.^12^ Thus, in PFE, an initial eruption pathway is seemingly established through PTH1R function in the coronal enamel organ but subsequent movement towards the occlusal plane is disrupted through loss of receptor function in the dental follicle. PFE is difficult to manage clinically because the teeth generally respond to orthodontic traction with ankylosis and disruption to the remaining occlusion, often taking place with prolonged traction (Fig. 3). The local restoration of PTHrP signalling in the dental follicle of these teeth would therefore seem to be an interesting possible future strategy to encourage normal eruption of these teeth in association with orthodontic traction.

Clinical management of PFE is dependent upon severity and the extent to which the dentition is affected. Orthodontic traction will result in ankylosis; although, some limited tooth movement may be achievable before this takes place. For milder presentations of infraocclusion, a decision may be made to accept a lateral open bite or attempt limited movement to improve it. Alternatively, prosthodontic restoration can restore the affected tooth or teeth into occlusion. For moderate or more severe cases, surgical intervention using segmental osteotomy and immediate elastic traction^24^ or distraction osteogenesis^25^^,^^26^ to level the occlusal plane have been described. Affected teeth may also be extracted and either orthodontic space closure or prosthetic replacement considered.

## Cleidocranial dysplasia

Cleidocranial dysplasia is an autosomal dominant skeletal dysplasia associated with defective bone ossification and severe dental anomalies; specifically, prolonged retention of the primary dentition, failure of eruption and multiple supernumerary teeth affecting the successional dentition^27^ (Table 1). The first permanent molars often erupt but eruption of the successional incisor and premolar dentition is highly variable. Mutations in the runt-related transcription factor RUNX2 have been identified as the cause of cleidocranial dysplasia (CCD) in most cases.^28^^,^^29^ This gene encodes a transcription factor essential for the terminal differentiation of osteoblasts. Mice generated with targeted disruption in *Runx2* have a complete absence of bone formation and they die at birth.^30^^,^^31^

The molecular mechanisms underlying the dental defects seen in CCD are also poorly understood; however, *Runx2* is widely expressed in odontogenic mesenchyme and interestingly, tooth development arrests at the bud stage in *Runx2* mutant mice.^32^^,^^33^^,^^34^^,^^35^ Investigating the supernumerary phenotype in humans has been compounded to some extent by the arrested tooth development seen in *Runx2* mutant mice. However, there is some evidence that Wingless-related integration site (Wnt) signalling activity is required in odontogenic mesenchyme for the suppression of successional tooth formation and Runx2 may be involved in suppressing the activity of Wnt inhibitors in these regions as part of this process.^36^ This suggests that, in human tooth development, elevated Wnt signal levels in the mesenchymal component of the developing dentition might be the basis of continued successional tooth formation taking place in CCD subjects.

**Fig. 4:**
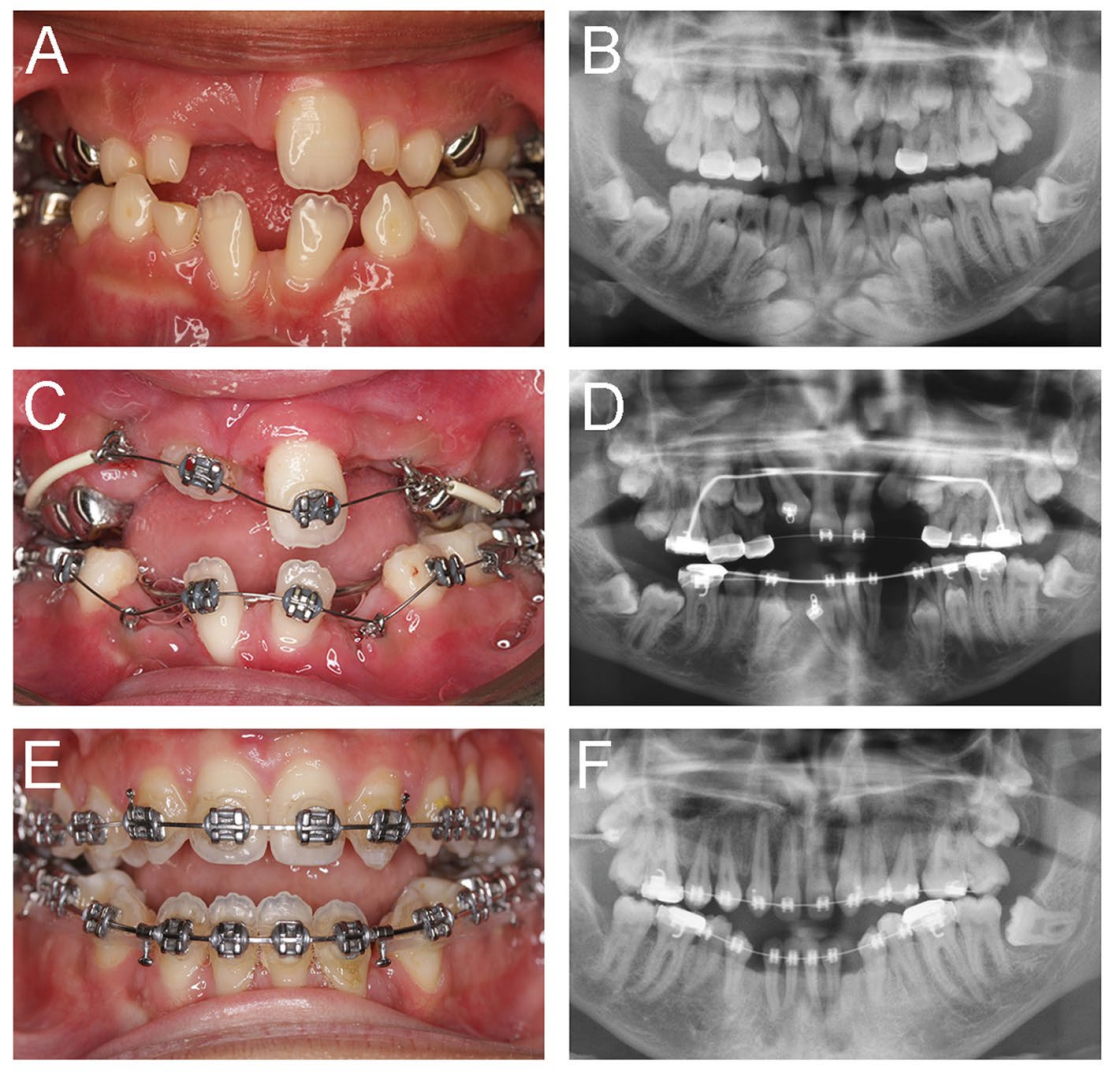
Management of CCD from the age of 10-18 years. a, b) A common feature of CCD is a failure of progression in dental development. In this case, there were four supernumerary teeth in the anterior mandible and impaction of the permanent canine teeth, while in the maxilla, there was impaction of several incisor teeth and a combination of tooth agenesis (22) and multiple supernumerary (supplemental fourth molars) teeth. c, d) The patient underwent multi-staged surgical interventions to extract supernumerary teeth and expose and bond impacted teeth, combined with the use of traction mediated by fixed orthodontic appliances to encourage eruption and establishment of the permanent dentition. e, f) At the age of 18 years, there was still a significant anterior open bite, which will be corrected with a bimaxillary osteotomy

The eruption phenotype in CCD is also poorly understood,^37^ but in contrast to PFE, while affected teeth in CCD rarely demonstrate eruption into the oral cavity, they often respond well to the application of orthodontic traction (Fig. 4). Is the inherent bone phenotype seen in these patients responsible for the eruption defect or is there a more local mechanism associated with the teeth themselves? Certainly, the dental phenotypes are thought to be distinct and multiple supernumerary teeth are not the cause of the failed eruption that is often seen. *RUNX2* is expressed in the dental follicle^38^ and there is evidence that mechanisms of bone formation and resorption are both impaired in the dental follicles of CCD patients through disrupted RANKL/RANK/OPG signalling.^39^^,^^40^ *Runx2* heterozygous mutant mice have delayed tooth eruption and reduced numbers of osteoclasts, which suggests that impaired alveolar bone resorption is an important mechanism in CCD.^41^ Indeed, it has recently been demonstrated that RUNX2 can directly regulate osteoclastogenesis,^37^ despite earlier reports to the contrary.^42^

## Conclusions

Understanding the biological basis of normal human tooth eruption mechanisms supports the accurate diagnosis of local and systemic eruption disorders and informs potential strategies to prevent or intercept these conditions. The continued identification of important loci and signalling pathways will aid these advancements and increasingly facilitate genetic testing, early diagnosis, and potentially preventive management of these conditions.
